# Contrasting In Vitro Apatite Growth from Bioactive Glass Surfaces with that of Spontaneous Precipitation

**DOI:** 10.3390/ma11091690

**Published:** 2018-09-12

**Authors:** Yang Yu, Zoltán Bacsik, Mattias Edén

**Affiliations:** Department of Materials and Environmental Chemistry, Stockholm University, SE-106 91 Stockholm, Sweden; yang.yu@mmk.su.se (Y.Y.); zoltanb@mmk.su.se (Z.B.)

**Keywords:** bioactive glass, biomimetic mineralization, apatite growth mechanism, simulated body fluid, quantification of apatite content, infrared spectroscopy, X-ray diffraction

## Abstract

Body-fluid-exposed bioactive glasses (BGs) integrate with living tissues due to the formation of a biomimetic surface layer of calcium hydroxy-carbonate apatite (HCA) with a close composition to bone mineral. Vast efforts have been spent to understand the mechanisms underlying in vitro apatite mineralization, as either formed by direct precipitation from supersaturated solutions, or from BG substrates in a simulated body fluid (SBF). Formally, these two scenarios are distinct and have hitherto been discussed as such. Herein, we contrast them and identify several shared features. We monitored the formation of amorphous calcium phosphate (ACP) and its crystallization into HCA from a Na${}_{2}$O–CaO–SiO${}_{2}$–P${}_{2}$O${}_{5}$ glass exposed to SBF for variable periods out to 28 days. The HCA growth was assessed semi-quantitatively by Fourier transform infrared spectroscopy and powder X-ray diffraction, with the evolution of the relative apatite content for increasing SBF-exposure periods evaluated against trends in Ca and P concentrations in the accompanying solutions. This revealed a sigmoidal apatite growth behavior, well-known to apply to spontaneously precipitated apatite. The results are discussed in relation to the prevailing mechanism proposed for in vitro HCA formation from silicate-based BGs, where we highlight largely simultaneous growth processes of ACP and HCA.

## 1. Introduction

Calcium hydroxyapatite (“apatite”; Ca${}_{5}($PO${}_{4}{)}_{3}$OH) is a naturally occurring mineral, whose carbonate-substituted form constitutes the inorganic part of mammalian bone and tooth [1]. Such a biomimetic hydroxy-carbonate apatite (HCA) phase forms also at the surface of a silicate-based *bioactive glass* (*BG*) on its exposure to body fluids [2,3]. The bone-bonding feature of BGs renders them suitable for bone grafting in periodontal, orthopedic, and maxillofacial surgery, where the “45S5 Bioglass” (with stoichiometry 24.6Na${}_{2}$O–26.7CaO–46.1SiO${}_{2}$–2.6P${}_{2}$O${}_{5}$) developed by Hench and co-workers has been in clinical use for decades [2,3].

Owing to its pivotal importance for biomineralization, numerous kinetic and thermodynamic aspects of in vitro apatite formation have been investigated extensively by examining spontaneous calcium phosphate (*CaP*) precipitation from supersaturated solutions of Ca and phosphate species [4,5,6,7,8,9,10,11,12,13,14,15,16,17,18,19]. It is well known that HCA generally forms via a precursor of amorphous calcium phosphate (ACP) according to a *sigmoidal* growth behavior, characterized by an “*induction*” period before the start of apatite crystallization, which then proceeds rapidly during the “*proliferation*” stage, followed by a slow “*maturation*” interval during which the HCA crystallinity is enhanced, whereas its amount is only marginally increased [4,5,6,7]. By “ACP”, we henceforth mean an amorphous CaP phase with a stoichiometry close to Ca${}_{3}$(PO${}_{4}$)${}_{2}$ and comprising minor (and variable) HPO${}_{4}^{-}$ contents and water molecules [7,20,21,22].

While the phenomenological CaP growth-characteristics are well understood, the precise details of the ACP→HCA conversion remains debated: early studies generally favored a dissolution–precipitation mechanism [5,10,11,12], whereas a solid-to-solid transformation [23,24] is advocated by many recent studies that moreover suggest HCA crystallization from the *interior* of the ACP particles [13,14,15]; yet, this topic lacks a clear consensus, where a surface-mediated crystallization is often favored [7,17,18,25,26]. For solutions with pH ≳ 9.5, ACP represents the *sole* precursor phase [11,12], whereas apatite crystallization in less alkaline solutions occurs via intermediate precursors roughly according to Ostwald’s step rule. Around the physiological pH = 7.4, the ACP→HCA transformation is reported to proceed via phases close to the octacalcium phosphate (OCP; Ca${}_{8}$$({\mathrm{HPO}}_{4}{)}_{2}($PO${}_{4}{)}_{4}\cdot 5$H${}_{2}$O) composition [6,7,9,16]. Habraken et al. [16] presented a detailed in vitro mechanism, demonstrating that the embryos of “ACP” constitute prenucleation [Ca(HPO${}_{4}$)${}_{3}$]${}^{4-}$ clusters in the solution, which evolve by further complexation with Ca${}^{2+}$ into apatite via OCP-like intermediates.

As for direct CaP precipitation, many studies have sought to unveil the in vitro HCA formation processes from silicate-based BGs [2,27,28,29,30,31,32,33,34,35,36,37,38,39,40,41,42,43], as well as their relationship to the glass composition and structure [2,3,29,40,43,44,45,46,47,48,49,50,51,52,53,54,55]. Here the glass is typically immersed in an acellular *simulated body fluid* (*SBF*) solution that matches the concentrations of inorganic species in human plasma [56,57]. The rate of apatite formation is often referred to as the “in vitro bioactivity”, which for silicate-based BGs has been shown to correlate well with their in vivo bone-bonding properties [58].

To rationalize the main stages of HCA formation from melt-prepared Na${}_{2}$O–CaO–SiO${}_{2}$–P${}_{2}$O${}_{5}$ BGs—or limiting ternary/binary glasses thereof—in (simulated) body fluids, Hench proposed a five-step sequence [2], henceforth dubbed the “*Hench mechanism*” (HM) and discussed further in Section 4.3. It comprises initial reactions at the glass surface (steps 1–3), followed by formation of an amorphous CaO–P${}_{2}$O${}_{5}$-rich film/layer (stage 4) and its (as proposed) *subsequent* crystallization into HCA (stage 5) [2]. Notably, much of the literature on apatite formation from BGs is overall vague as to the details of this amorphous “CaO–P${}_{2}$O${}_{5}$-rich film” [2], whose precise chemical/structural nature is challenging to characterize experimentally. Yet, recent solid-state ${}^{1}$H/${}^{31}$P NMR studies [36,37,38,39,40,44] of the amorphous portion of the in vitro-formed CaP surface layer (a.k.a. the “CaO–P${}_{2}$O${}_{5}$-rich film”) reveal very similar NMR parameters with those reported from ACP precipitated from calcium phosphate solutions [20,21,22,59,60]. Notwithstanding that many researchers in the BG community do identify the “CaO–P${}_{2}$O${}_{5}$-rich film” with “ACP” [37,38,39,40,41,42,44,45,46,47,61], this salient feature appears not to be widely recognized.

Herein, we examine the ACP/HCA growth from a melt-prepared Na${}_{2}$O–CaO–SiO${}_{2}$–P${}_{2}$O${}_{5}$ glass powder immersed in SBF for variable periods out to 28 days. The relative HCA content was assessed semi-quantitatively by transmission mode Fourier transform infrared (FTIR) spectroscopy using an internal standard. The results are discussed in conjunction with trends from powder X-ray diffraction (XRD) data together with measured pH-values and concentrations of Ca and P in the accompanying solutions: they reveal that heterogenous CaP nucleation at the SBF-exposed glass surface exhibit *identical* sigmoidal growth characteristics as those for apatite formed directly from supersaturated solutions [4,5,6,7,8,9,10,11,12,13,14,15,16,17,18,19]. We contrast these two *formally* distinct HCA formation scenarios and discuss some of their qualitative similarities, many of which are apparent in the experimental data of earlier reports, although they have remained unrecognized, presumably mainly stemming from the qualitative nature of these studies. We also examine the HM further, where we highlight overall *simultaneous rather than sequential* formations of ACP and HCA from BG surfaces.

A note on terminology: we employ the terms “HCA” and “apatite” interchangeably, noting that carbonate-substituted hydroxyapatite (i.e., HCA) is always obtained from BGs in SBF solutions [2], whereas the term “H(C)A” is used when contrasting/discussing our results in relation to the literature on spontaneously precipitated apatite, where the experimental conditions normally ensure a carbonate-free product.

## 2. Materials and Methods

### 2.1. Glass Preparation and SBF Testing

A glass of 24.26Na${}_{2}$O–26.33CaO–45.41SiO${}_{2}$–4.00P${}_{2}$O${}_{5}$ stoichiometry was prepared by a standard melt-quench procedure from ball-mixed precursors of 2.608 g SiO${}_{2}$ (99.99% purity), 2.053 g Na${}_{2}$CO${}_{3}$ (99.99%), and 2.519 g CaCO${}_{3}$ (99.9%) from ChemPur (Karlsruhe, Germany), and 0.918 g NaH${}_{2}$PO${}_{4}$ (99.99%) from Merck (Darmstadt, Germany). The precursor mixture was decarbonated at 950 ${}^{\circ }$C in a Pt crucible for 2.5 h in an electric furnace, and was subsequently melted at 1450 ${}^{\circ }$C for 2.5 h, followed by rapid quenching by immersing the bottom of the crucible in water. The glass homogeneity and absence of significant crystalline impurities (≲1%) was confirmed by scanning electron microscopy in back-scatter mode (JSM-7000F; JEOL Ltd., Tokyo, Japan), and powder XRD (see Section 2.3), respectively, as described in detail in our previous work [49,50,62].

The pristine glass is henceforth denoted by “BG45${}_{4.0}$”, where the numbers “45” and “4.0” represent the respective (nominal) SiO${}_{2}$ and P${}_{2}$O${}_{5}$ contents in mol%. This BG composition is associated with a silicate network connectivity [51] of ${\bar{N}}_{\mathrm{BO}}^{\mathrm{Si}}=2.3$, which together with the relatively high P content ensures a high in vitro bioactivity [49,51,52,53]. Note that the composition of the glass is similar (yet distinct) to that of 45S5 [2].

The in vitro apatite formation from the BG45${}_{4.0}$ glass was monitored in an SBF solution [56], prepared by dissolving ACS grade NaCl, NaHCO${}_{3}$, KCl, K${}_{2}$HPO${}_{4}$·3H${}_{2}$O, MgCl${}_{2}$·6H${}_{2}$O, CaCl${}_{2}$, and Na${}_{2}$SO${}_{4}$ in water (ENSURE, Merck) to give concentrations of [Ca] = 2.5 mM and [P] = 1.0 mM [56]. The pH-value was adjusted to 7.40 by using tris hydroxymethylaminomethane and 1.0 M HCl. The SBF testing employed the procedure of Kokubo et al. [56], except for using glass powders (50–100 μm particles) instead of bulk pieces, where 250 mg of the powder was dispersed into 250 mL of SBF solution in a 500 mL polypropylene container, which is equivalent to a mass concentration of 1.00 g/L. The container was incubated in a water bath (36.5 ± 0.2 ${}^{\circ }$C) for a given SBF immersion period ($\tau_{\mathrm{SBF}}$), whereupon 50 mL of the supernatant was extracted for subsequent pH and cation concentration measurements, whereas the remaining solution was passed through a glass microfiber paper (0.7 μm). To quench the surface reactions and achieve a rapid drying without NaCl precipitation [63], the solid phases were washed consecutively with deionized water, ethanol, and acetone.

The SBF immersion procedure described above was performed twice for each soaking period of $\tau_{\mathrm{SBF}}=${4, 8, 12, 24, 72, 336, 672} h, with the two longest intervals corresponding to 14 days and 28 days, respectively. For each $\tau_{\mathrm{SBF}}$-value, the associated measured properties (pH-values, Ca and P concentrations, IR spectra, and IR-derived apatite contents) were averaged to yield the data discussed below for each SBF-exposed specimen, which is onwards denoted as BG45${}_{4.0}$–$\tau_{\mathrm{SBF}}$, with $\tau_{\mathrm{SBF}}$ specified in hours.

### 2.2. Measurements of pH-Values and Ion Concentrations

The pH values and the Ca concentrations in the solutions accompanying each BG45${}_{4.0}$–$\tau_{\mathrm{SBF}}$ powder were measured at 35 ${}^{\circ }$C and 25 ${}^{\circ }$C, respectively, using a Metrohm meter (827 pH Lab; Herisau, Switzerland) equipped with a Ca-selective electrode. A constant ionic strength was arranged by adding 1.00 M KCl to each solution (1:1 volume ratio). Phosphorus concentrations were determined with an UV-Vis spectrometer (Lambda 19; Perkin Elmer, Waltham, MA, USA) by measuring the absorption of “Mo Blue” (λ=880 nm), formed by mixing the solution with ascorbic acid (99.7%; Merck), 50% H${}_{2}$SO${}_{4}$ (VWR), as well as (NH${}_{4}$)${}_{2}$MoO${}_{4}$ (99%) and antimony potassium tartrate (C${}_{8}$H${}_{4}$K${}_{2}$O${}_{12}$Sb${}_{2}\cdot x$H${}_{2}$O; 98%) from Alfa Aesar (Haverhill, MA, USA) [64]. The absorbance was measured (400–1300 nm) in a cuvette with 1 cm light path. Interferences in the [P] determinations from silicate ions (stemming from the glass dissolution) were minimized by employing the procedure of ref. [65]. pH, [Ca] and [P] values were measured in triplicate for each of the two independent BG45${}_{4.0}$–$\tau_{\mathrm{SBF}}$ preparations.

### 2.3. Powder XRD and FTIR Experiments

XRD data were recorded from BG45${}_{4.0}$–$\tau_{\mathrm{SBF}}$ powders dispersed on a zero-background silica substrate, utilizing a PANalytical X’Pert PRO MPD diffractometer (Almelo, The Netherlands) equipped with an X’Celerator detector, Cu $K_{\alpha }$ radiation (λ=154.1 pm), and variable divergence slits. Each diffractogram was acquired over a 2θ range of 10–65${}^{\circ }$ for a total time-span of 3 h.

The apatite content of each BG45${}_{4.0}$–$\tau_{\mathrm{SBF}}$ specimen was quantified by transmission mode FTIR spectroscopy (Varian 610-IR Instrument, Palo Alto, CA, USA). Each IR spectrum was recorded over a 400–4000 cm${}^{-1}$ wavenumber range with a resolution of 2 cm${}^{-1}$. K${}_{3}$Fe(CN)${}_{6}$ (99%; Alfa Aesar) was used as internal standard [66], with 10.0 mg glass powder, 7.5 mg K${}_{3}$Fe(CN)${}_{6}$, and 3.000 g KBr (IR grade; Merck) mixed and pressed into a pellet (13 mm in diameter; 10${}^{4}$ kg load); these relative amounts of BG45${}_{4.0}$–$\tau_{\mathrm{SBF}}$ and KBr ensured a linear response between the IR absorbance and the HCA content.

By scaling all IR spectra to an equal intensity of the narrow band at 2117 cm${}^{-1}$ from the C≡N vibrations of K${}_{3}$Fe(CN)${}_{6}$ (see Figure S1), we ensured that the integrated IR intensity of the split bands in the region 522–660 cm${}^{-1}$ directly conveys the relative HCA content of the BG45${}_{4.0}$–$\tau_{\mathrm{SBF}}$ sample. In the absence of a discernible band-splitting, i.e., when no apatite was detected, a zero “HCA content” is reported. This procedure eliminates the dependence of the IR intensity on the thickness of the KBr pellet and offers quantitative *relative* apatite contents among the BG45${}_{4.0}$–$\tau_{\mathrm{SBF}}$ samples, as discussed further in ref. [53]. Each reported average HCA content and its accompanying uncertainty were obtained from two independent SBF tests (see Section 2.1); the IR spectrum of each BG45${}_{4.0}$–$\tau_{\mathrm{SBF}}$ specimen presented below is an average over those two outcomes.

## 3. Results

### 3.1. Apatite Formation Probed by XRD and FTIR

**Figure 1:**
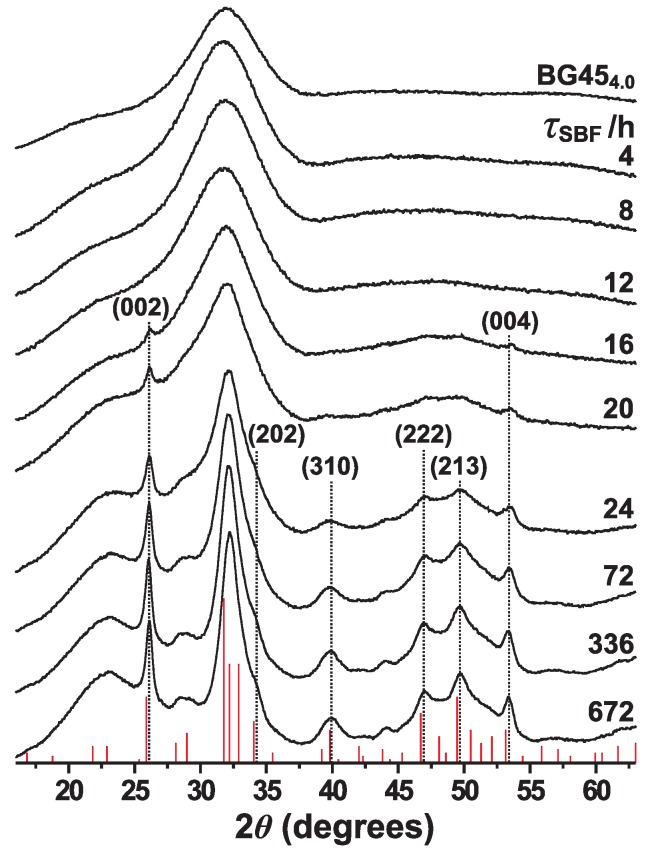
Powder XRD patterns recorded from the pristine BG45${}_{4.0}$ glass (top trace), as well as the SBF-exposed BG45${}_{4.0}$–$\tau_{\mathrm{SBF}}$ specimens, with the as-indicated SBF-soaking periods ($\tau_{\mathrm{SBF}}$) increasing from top to bottom. Also shown (red sticks) is a generic diffractogram representative of well-crystalline hydroxyapatite (International Centre for Diffraction Data: data-set 00-09-0432). The dotted vertical lines indicate a selection of Bragg peaks and their associated Miller indices.

Figure 1 shows powder XRD patterns obtained from the pristine BG45${}_{4.0}$ glass and the BG45${}_{4.0}$–$\tau_{\mathrm{SBF}}$ specimens with variable SBF-soaking intervals (4 h $⩽\tau_{\mathrm{SBF}}⩽672$ h), as well as a generic diffractogram representative of highly crystalline apatite. Consistent with its amorphous nature, the pristine glass produce no sharp peaks. Also all diffractograms recorded from the SBF-exposed glass powders with $\tau_{\mathrm{SBF}}⩽$12 h remain very similar to that of the parent glass, thereby confirming the absence of crystalline phases. The emergence of HCA is evident after 16 h of SBF immersion, where the BG45${}_{4.0}$–16 h specimen reveals two minor Bragg peaks, whose corresponding 2θ angles are attributed to the (002) and (004) lattice planes of apatite; see Figure 1. The sharp peak associated with the (002) plane generally develops first among the Bragg peaks, thereby serving as a marker for the onset of apatite formation. As reflected by increasing diffraction intensities, the apatite content then grows for extended SBF-soaking out to $\tau_{\mathrm{SBF}}⩽72$ h. For SBF immersion periods beyond 24 h, the diffraction peaks reveal a modest growth but a slight narrowing, which is most transparent between 24–336 h and suggesting a slowly increased degree of structural ordering/crystallinity (as is more evident from the IR results discussed below). Yet, owing to the nanocrystalline character of the in vitro-grown HCA, its Bragg peaks remain markedly wider than those observed from well-ordered apatites.

**Figure 2:**
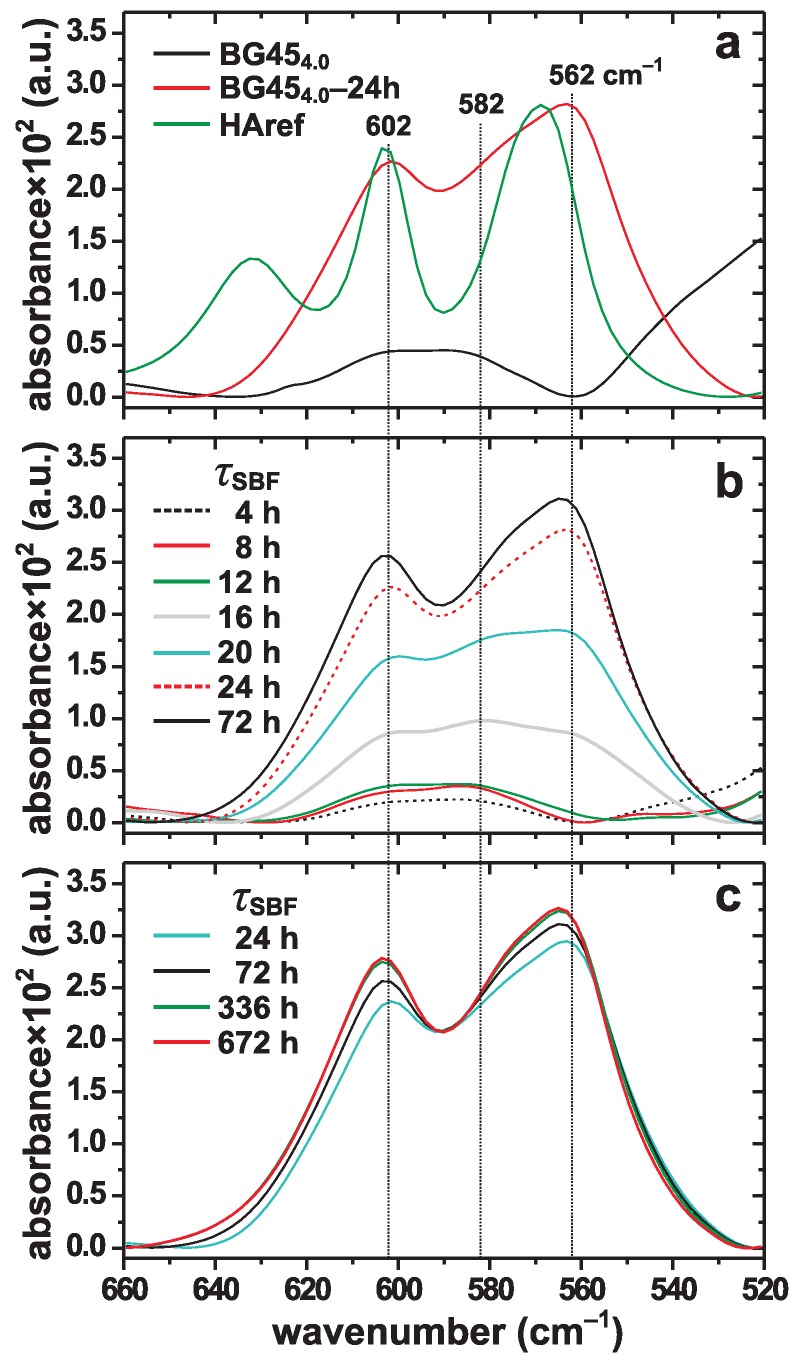
(**a**) FTIR spectra recorded from the BG45${}_{4.0}$ glass powder before and after SBF exposure for 24 h, shown together with the results from a highly crystalline hydroxyapatite reference powder (HAref); the relative intensities are plotted in arbitrary units (a.u.). The spectra are zoomed over the wavenumber range relevant for discriminating IR responses from PO${}_{4}^{3-}$ groups in disordered (BG45${}_{4.0}$) and ordered (BG45${}_{4.0}$–24 h and *HAref*) structural environments. (**b**,**c**) FTIR spectra from BG45${}_{4.0}$–$\tau_{\mathrm{SBF}}$ glass powders exposed to SBF for the as-indicated $\tau_{\mathrm{SBF}}$ periods. Note that the data are normalized relative to the integrated intensity of BG45${}_{4.0}$–72 h in (**b**), but to equal spectral intensities at 590 cm${}^{-1}$ in (**c**) to better convey the more pronounced band splitting that develops for increasing $\tau_{\mathrm{SBF}}$.

Figure 2a displays IR spectra recorded from powders of the BG45${}_{4.0}$ glass before and after SBF exposure ($\tau_{\mathrm{SBF}}=24$ h), together with results from a well-ordered polycrystalline hydroxyapatite reference sample (“*HAref*”). The spectra are zoomed over the 520–660 cm${}^{-1}$ wavenumber range, which is the relevant range for discriminating between orthophosphate groups in amorphous and ordered structural environments [48,54,61,67,68,69]; Figure S1 of the Supporting Information displays a selection of spectra over the full wavenumber range. The BG45${}_{4.0}$–24 h specimen reveals split bands around 562 cm${}^{-1}$ and 602 cm${}^{-1}$, which originate from the bending modes of P–O bonds [48,54,61,67,68] in H(C)A. Owing to the nanocrystalline nature and partial carbonate substitution in the apatite structure [2,67,68,69], these IR bands are broader than those observed from the *HAref* powder. In contrast, the PO${}_{4}^{3-}$ anions of the pristine BG45${}_{4.0}$ glass structure only produce a weak and very broad band centered around 590 cm${}^{-1}$, characteristic of orthophosphate groups in amorphous structures, such as from Na–Ca–Si–P–O glasses and ACP [48,54,61,67,68]; the latter is revealed by the IR spectra obtained for short SBF exposure periods $\tau_{\mathrm{SBF}}⩽12$ h (Figure 2b).

In full accordance with the powder XRD results (Figure 1), the IR spectra of Figure 2b give no evidence for HCA formation during the first 12 h of SBF immersion, while the overall low IR intensities observed also implies insignificant ACP contents in all BG45${}_{4.0}$–$\tau_{\mathrm{SBF}}$ specimen with $\tau_{\mathrm{SBF}}⩽12$ h. However, a significant IR-intensity boost is observed at $\tau_{\mathrm{SBF}}=16$ h: while remaining broad, these bands reveal maxima around 562 cm${}^{-1}$ and 602 cm${}^{-1}$, *as well as* ≈582 cm${}^{-1}$, thereby evidencing substantially increased amounts of *both* ACP and HCA, which moreover unambiguously co-exist. The further growth of primarily HCA (at the expense of ACP) is evident at $\tau_{\mathrm{SBF}}=20$ h, whereas the HCA-associated IR bands dominate for all longer SBF immersion periods. Most of the minor remnants of ACP are attributable to surface layers of the nanocrystalline HCA particles, as discussed in [21,22,39].

**Figure 3:**
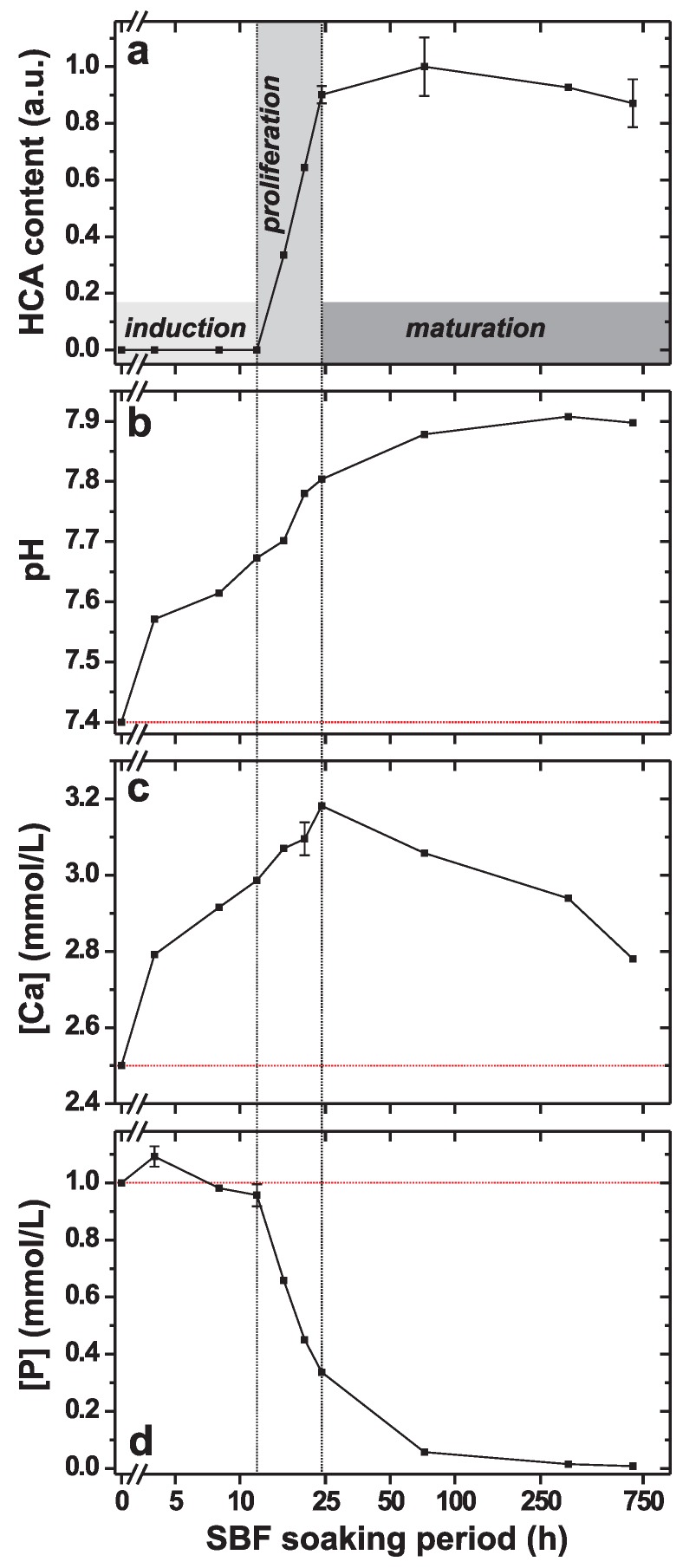
(**a**) IR-derived relative HCA contents in BG45${}_{4.0}$–$\tau_{\mathrm{SBF}}$ specimens plotted against $\tau_{\mathrm{SBF}}$. (**b**–**d**) Results of (**b**) pH, (**c**) [Ca] and (**d**) [P] measured in the accompanying solutions. Note the usage of a logarithmic ($\log_{10}$) horizontal scale. The dotted vertical lines and the shaded domains highlight the corresponding induction, proliferation, and maturation stages normally discussed in the context of spontaneous apatite formation, while red dotted horizontal lines in (**b**–**d**) indicate the respective values of the pH, [Ca] and [P] in the pristine SBF solution (i.e., for $\tau_{\mathrm{SBF}}=0$). Note that error bars are only displayed when outside of the symbols. The largest relative amount of HCA (observed at $\tau_{\mathrm{SBF}}$ = 72 h) corresponds to 29 ± 3 wt% HCA out of all solid phases.

The relative HCA content of each BG${}_{4.0}$(2.3)–$\tau_{\mathrm{SBF}}$ specimen with $\tau_{\mathrm{SBF}}>12$ h was determined by integrating the IR intensity across the 522–660 cm${}^{-1}$ wavenumber range of each spectrum displayed in Figure 2b,c. We comment that due to the (essentially) *simultaneous* formation of ACP and HCA occurring between 12–20 h, this quantification procedure is prone to slightly overestimating the apatite content in this $\tau_{\mathrm{SBF}}$ regime [53]. Figure 3a plots the resulting relative HCA contents against $\tau_{\mathrm{SBF}}$ over 672 h (28 days) of SBF soaking. As commented above, the ACP→HCA crystallization starts rapidly between 12–16 h, with HCA first detected at $\tau_{\mathrm{SBF}}=16$ h (in full agreement with the XRD results), followed by a steep and nearly linear increase against $\tau_{\mathrm{SBF}}$ out to 24 h, at which the apatite content reached 90% out of the global maximum observed at 72 h.

For SBF immersion periods beyond 24 h, the HCA formation rate slows down markedly, with an apparent slight decrease in the apatite content observed for $\tau_{\mathrm{SBF}}>72$ h (as obtained from IR spectroscopy). This may reflect an inhibited growth due to a low amount of phosphate species in the solution; see Section 3.2 and refs. [40,70,71]. Yet, Figure 2c reveals progressively developed IR-band splittings from $\tau_{\mathrm{SBF}}$ = 24 h to $\tau_{\mathrm{SBF}}$ = 336 h that may reflect an enhanced structural ordering or an improved crystallinity of the apatite phase, in agreement with the concurrent (although minor) Bragg-peak narrowing in Figure 1 during the same SBF-immersion intervals.

### 3.2. Trends of pH, [Ca] and [P] Solution Parameters

When a *bioactive* glass is exposed to SBF, rapid ion-leaching/proton-exchange processes are observed during the first few hours of immersion [2,41,48,54,61,68], leading to a concomitant increase in the pH value of the solution. Indeed, Figure 3b,c reveals growing pH-values and Ca concentrations for increasing SBF-soaking periods up to 24 h. After reaching a maximum at $\tau_{\mathrm{SBF}}=24$ h, the [Ca] values drop for extended SBF exposure out to 28 days (Figure 3c), accompanied by a continuously growing pH of the solution towards an asymptotic value ≈7.9 (Figure 3b).

In contrast with the pH and [Ca] values of the solution, the P concentrations plotted in Figure 3d strongly *correlate* with the extent of HCA formation, as also reported previously [48,53,61,68,72,73]. During the first 12 h of SBF soaking, [P] remains almost constant around the value initially observed in the SBF solution, although a slight increase is discernible at $\tau_{\mathrm{SBF}}=4$ h, followed by a decrease between 4–8 h; these [P] alterations are attributed to the consecutive processes of glass dissolution and ACP formation. Yet, the IR spectra of Figure 2a and its respective relative HCA contents of Figure 3a reveal that the latter process is *limited* up to $\tau_{\mathrm{SBF}}\approx 12$ h. In contrast, the CaP layer formation accelerates significantly throughout the SBF-soaking interval of 12–24 h, during which the P concentration diminishes steeply and concurrently with the coincident ACP and HCA growth processes, with the latter dominating from ≈20 h (see Section 3.1). After 24 h of SBF-immersion, the P concentration amounts to ≈33% of the value in the pristine SBF solution, whereupon [P] slowly but progressively diminishes for growing $\tau_{\mathrm{SBF}}$, such that only ≈5% of its initial value is observed after 72 h.

The strong (inverse) correlation between the extent of apatite formation (Figure 3a) and the P concentration of the accompanying solution (Figure 3d) is worth underscoring, where identification of the steep decrease in [P] is a convenient and reliable marker for the *onset* of apatite formation; a *qualitative* relationship between the apatite-formation degree and the P concentration of the solution is well-documented in the literature [48,72,74,75]. As discussed by Yu et al. [53] from a more general viewpoint than solely for identifying the start of HCA formation, the readily measured P concentration offers a straightforward *semi-quantitative* assessment of the relative HCA amount formed from BGs with different compositions, thereby permitting convenient in vitro bioactivity screenings without requiring techniques to *directly* determine the apatite content.

## 4. Discussion

### 4.1. Similarities between Apatite Formation by Direct Precipitation and from Glass Surfaces

This section highlights several apparent similarities between apatite formation by spontaneous precipitation from Ca/P-bearing solutions with HCA production at BG surfaces in SBF/buffered water. These two scenarios are *formally* distinctly different but have hitherto not been contrasted. Spontaneous CaP precipitation starts by a *homogeneous* ACP formation from typically relatively concentrated solutions with [P] ≲ [Ca] ≈ 10 mM. At a BG surface, on the other hand, CaP is nucleated *heterogeneously* at low supersaturation conditions in an SBF solution ([Ca] = 2.5 mM; [P] = 1.0 mM [57]) that is stable for weeks without precipitation; indeed, inherent to its potential biomedical applications, the emergence of HCA from a given glass substrate should *solely* reflect its inherent (in vitro) bioactivity.

Notwithstanding these formal differences, a key finding herein is that CaP formed by heterogenous nucleation at SBF-exposed BGs surfaces exhibits *identical* sigmoidal growth characteristics as those well-established for spontaneous H(C)A precipitation [4,5,6,7], which comprise three stages of “induction”, “proliferation”, and “maturation”; see Figure 1 of ref. [5] for representative examples. The sigmoidal signatures are evident from the relative HCA contents observed for increasing SBF immersion intervals in Figure 3: after a 12 h induction period, the proliferation stage proceeds between 12–24 h, whereupon exposure periods $\tau_{\mathrm{SBF}}>24$ h reflect the maturation interval. While the precise duration of each induction/proliferation step will depend on the degree of bioactivity, in turn dictated by the precise glass composition, the phenomenological sigmoid HCA growth appears to apply generally, as discussed further below.

Worth commenting is that a sigmoidal growth is anything but restricted to apatite formation, but is commonly observed for solution-mediated crystal nucleation/growth [76]. Yet, the identical apatite formation trends observed for BGs in SBF with those for direct precipitation appear to be unrecognized by the research community, presumably mainly because in vitro-bioactivity assessments of BGs to date are qualitative rather than quantitative, and partially because they solely targeted determinations of the in vitro bioactivity of the glass, deduced from the time required for initiation of HCA formation, i.e., the induction period [54,75,77,78]. The sigmoidal apatite growth was also unrecognized in one of our recent studies [40], where HCA formation from SBF-exposed CaO–SiO${}_{2}$–P${}_{2}$O${}_{5}$ mesoporous bioactive glasses (MBGs [46,55]) was quantified by powder XRD and ${}^{31}$P NMR: notably, the HCA contents plotted against $\tau_{\mathrm{SBF}}$ in Figure 6 of ref. [40] for three MBGs with different {Ca, Si, P} contents manifested the sigmoid-type functionality for one composition (“S58”), whereas the other two MBGs revealed a continuous apatite growth throughout 30 days of SBF soaking, thereby obscuring a clear discrimination between the proliferation and maturation stages.

In the following, we discuss each consecutive induction, proliferation, and maturation stage, first outlining its salient features established in the context of spontaneous CaP precipitation, followed by comments on the similarities/distinctions to the case of apatite formed at BG surfaces.

#### 4.1.1. Induction Period

The induction period comprises all mechanistic steps up to which HCA, or any structurally ordered CaP precursor thereof, is first observed (see Figure 3). It is the *sole* apatite-formation stage assessed and discussed in the context of SBF-exposed BGs. The main event of the induction interval involves formation of ACP, which is generally accepted as the initial precursor of apatite when formed directly from aqueous solutions with pH >6.5 and each (total) concentration of Ca and phosphate species exceeding a few mM [8,9]. However, in the “low supersaturation” regime of (initial) concentrations of [Ca] ≲ 2 mM and [P] ≲ 1 mM, there is no evidence for ACP or any other precursor phase [8,9], but poorly ordered apatite (“apatite dots”) is argued to form directly [8].

In the context of spontaneous precipitation, the net apatite-formation rate is known to depend foremost on the temperature of the solution, with *each* induction, proliferation, and maturation stage becoming accelerated at elevated temperatures [5,6,10]. Moreover, the induction period lengthens for a decrease in either of the Ca or P concentration, or for increasing pH across the range 6.5≲ pH ≲9.5, whereas it shortens when the pH is increased further [5,7,10,11,12]. Varying the pH or temperature is not relevant for in vitro testing of biomaterials, where the closest emulation of physiological conditions (pH = 7.4; 37 ${}^{\circ }$C) is desirable [56,57]. Yet, estimated induction periods for HCA formation from various silica gels in modified SBF solutions that comprised higher [Ca] and/or [P] values [75,77] than human plasma [57] revealed the same pH/temperature trends as for direct apatite precipitation, except that a minor pH reduction from 7.4 to 7.0 *lengthened* the induction period [75,77]; nevertheless, a recent in vitro study of “45S5 Bioglass” confirmed the expectations of a *shortened* induction period for decreasing pH [78], as for spontaneous precipitation.

#### 4.1.2. Proliferation Period

Starting at the onset of H(C)A formation, the “proliferation” interval is responsible for the majority (typically >80%) of the apatite growth [5,7,10,11,12]. For instance, Figure 3a shows that the BG45${}_{4.0}$–24 h specimen comprises 90% of the globally largest apatite amount observed (at $\tau_{\mathrm{SBF}}$ = 72 h). As reflected directly by the mere phenomenological sigmoid growth curve of Figure 3a, the heterogeneous HCA formation from BG surfaces is “autocatalytic”, meaning that it accelerates concomitantly with the number of apatite crystals present. The autocatalytic growth signature is well-documented for spontaneous apatite precipitation [4,5,6,7], but this shared property of heterogenous CaP nucleation from glass/silica-gel substrates in aqueous solutions has not yet been highlighted or discussed. For highly supersaturated solutions, the rapid apatite crystallization during the proliferation stage is accompanied by pronounced drops in the values of pH, [Ca] and [P] [5,6,7,15]. While the proliferation period associated with HCA formation from the SBF-exposed BG in Figure 3d indeed reveals a significant reduction of [P], the ion-exchange processes between Na${}^{+}/$Ca${}^{2+}$ species of the glass with protons from the SBF generally manifest continuously *increasing* pH and [Ca] values, as witnessed by Figure 3b,c.

Notably, while Figure 3 unambiguously evidences the “autocatalytic” HCA-formation signature applies to heterogenous CaP nucleation at the BG surface, the results strongly suggest largely *simultaneous* (rather than sequential) ACP formation and ACP→HCA transformation processes from the SBF-soaked BG (see Section 4.2): during the induction period ($\tau_{\mathrm{SBF}}⩽12$ h), the IR spectra of Figure 2b manifest very weak signal intensities, while the corresponding P concentrations change marginally. Altogether, these observations are consistent with a minor ACP formation for all immersion intervals up to 12 h. However, the IR band from ACP persists at $\tau_{\mathrm{SBF}}$=16 h, now co-existing with the HCA-characteristic split bands (520–660 cm${}^{-1}$); these IR spectral features coincide with an intensity boost, thereby evidencing significantly increased ACP and HCA contents of the BG45${}_{4.0}$–16 h specimen, as further corroborated by the concomitant reduction in [P]; see Figure 3d. While these IR-intensity/[P] trends prevails *throughout* the proliferation period (12 h < $\tau_{\mathrm{SBF}}$ ⩽24 h), the ACP→HCA transformation process gradually emphasizes during the longer SBF-soaking intervals. Along our observations for heterogenous CaP nucleation/growth events at BG surfaces, recent in vitro CaP precipitation reports using solutions at physiologically relevant Ca and P levels suggest that *both* ACP and H(C)A growth processes occur largely simultaneously [18,26]. Yet, at high(er) supersaturation conditions, a substantial ACP formation is generally anticipated prior to the start of apatite crystallization.

#### 4.1.3. Maturation Period

Apatite precipitation studies demonstrate that the maturation period is characterized by a rather modest increased *amount* of apatite and mainly a progressively enhanced *structural ordering* of the apatite crystallites (primarily by Ostwald ripening) [4], accompanied by a concurrent alteration in the stoichiometric Ca/P ratio [6,7]. These features of the nanocrystalline HCA formed at the BG45${}_{4.0}$ surface are witnessed by the IR and XRD data in Figure 1 and Figure 2c (see Section 3.1). The maturation accelerate for increasing temperature, along expectations of an improved degree of crystallinity obtained at elevated temperature [6,10].

### 4.2. Further Support for Coincident Net ACP/HCA Growth

Both the evolution of the P concentration in the solution (Figure 3d) and the corresponding FTIR spectra (Figure 2b) across the “induction” ($\tau_{\mathrm{SBF}}⩽12$ h) and “proliferation” (12 h$<\tau_{\mathrm{SBF}}$⩽24 h) periods are consistent with a *minor* ACP production during the former interval, but largely *simultaneous* ACP formation and ACP→HCA crystallization events throughout the proliferation, during which a majority of all CaP phases form. This feature revises the HM by partially merging its two last steps (4 and 5), as discussed further in Section 4.3. Although this property is not discussed in previous papers and consequently seems to have remained unnoticed, it appears to be quite general. This becomes evident by “data reinterpretation” of numerous reports of in vitro HCA formation from widely spanning amorphous phosphosilicate compositions in buffered water or SBF, encompassing glasses from the quaternary Na${}_{2}$O–CaO–SiO${}_{2}$–P${}_{2}$O${}_{5}$ system [42,48,67], and the limiting ternary Na${}_{2}$O–SiO${}_{2}$–P${}_{2}$O${}_{5}$ [48] and CaO–SiO${}_{2}$–P${}_{2}$O${}_{5}$ [72] systems, as well as for P-free Na${}_{2}$O–CaO–SiO${}_{2}$ [48], Na${}_{2}$O–SiO${}_{2}$ [41,48], and CaO–SiO${}_{2}$ [48,72] glasses; this feature also applies for apatite formation from “silica gels” (hydrated SiO${}_{2}$) [75,79], which exhibit a low but non-negligible bioactivity.

The above-cited papers [41,48,67,72,75,79]—as well as many others—present (raw) IR spectra revealing features similar to those of Figure 2: no significant ACP signal was observed before the occurrence of a large intensity boost that coincided with the development of “split bands”, signifying the presence of HCA, as often further corroborated by XRD results. Moreover, the precise glass preparation (melt or sol–gel derived) and in vitro testing conditions do not appear to affect these outcomes significantly, with the same qualitative trends resulting from glass powders as well as monoliths. The spectroscopic data were often supplemented by measured P concentrations that typically showed a ≲ 30% consumption of the initial [P] value during the induction period, followed by a rapid reduction in [P] during the (herein identified) “proliferation” stage. Below, we single out three studies that are commented in more detail:

Ohtsuki et al. [72] soaked glasses with stoichiometric compositions 50CaO–50SiO${}_{2}$ and 50CaO–45SiO${}_{2}$–5P${}_{2}$O${}_{5}$ in SBF: both revealed a weak IR absorption from ACP but significantly higher intensities once the IR signatures of H(C)A emerged, which was accompanied by a clearly reduced P concentration [72]. We estimated from their [P] data that ≈30% of the total P reservoir was consumed during the induction period associated with the SBF-immersion of the 50CaO–50SiO${}_{2}$ glass, whereas [P] altered marginally throughout the induction interval associated with the P-bearing glass, whereupon the solution became fully depleted of phosphate ions during the subsequent apatite crystallization stage, along the results presented in Figure 3a,d. On the other hand, Pereira et al. [75] studied HCA growth from a silica gel (with 1.2 nm average pore size) exposed to SBF. Their IR data revealed a marginal ACP formation (accompanied by a [P] reduction of only a few percent during the induction period), whereas once the ACP→HCA crystallization was detected by IR spectroscopy, the P concentration dropped markedly. Moreover, Martin et al. [42] investigated apatite growth from “45S5 Bioglass” using surface-sensitive shallow-angle XRD. Their results clearly indicated that *both* ACP and HCA phases grew during 24–72 h of SBF exposure (although the authors did not comment on the aspect of simultaneous ACP/HCA formation processes).

We conclude that most literature reports of apatite formation from BG surfaces are indeed consistent with only a minor ACP production before the onset of HCA crystallization, followed by largely coincident ACP/HCA formation processes. Yet, there are a few exceptions [45,80] that reveal a significant ACP formation during the induction period. None of these results appear to stem from inappropriate SBF testing conditions, which are otherwise the primarily origin for apparent delays between the emergence of each ACP and HCA phase [40,43,53,61,70,71]. For instance, a too high glass loading in the aqueous medium may lead to a depletion of phosphate species in the solution due to a substantial Ca release from the glass, thereby resulting in the *apparent* sole presence of ACP due to a markedly retarded—or even quenched—ACP→HCA conversion, as discussed further in refs. [40,53,70,71].

### 4.3. Discussion on the Validity of the Hench Mechanism

**Figure 4:**
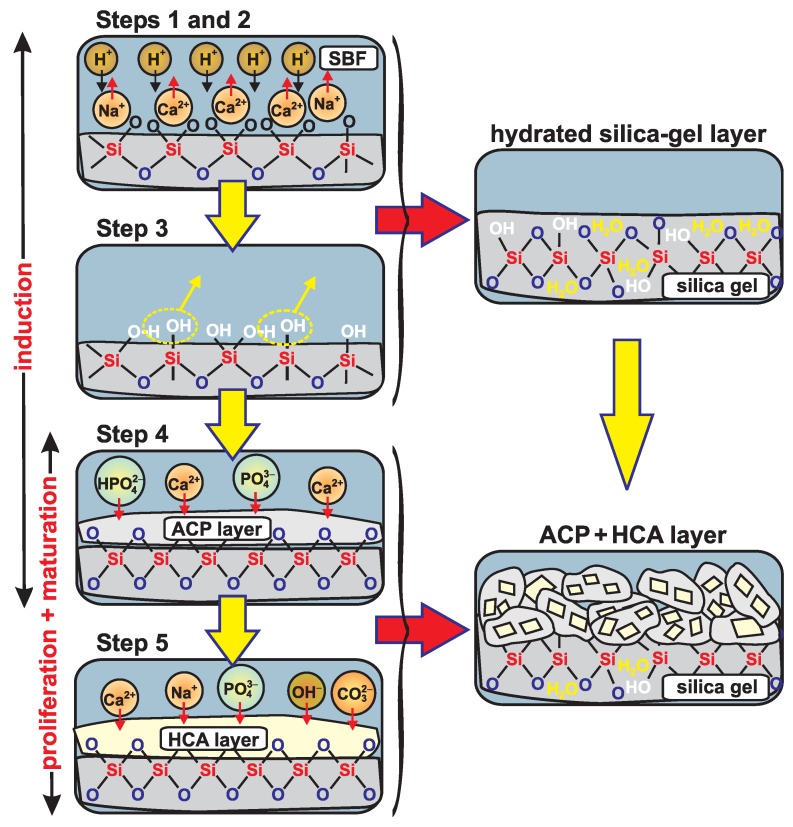
Schematic illustration of the Hench mechanism [2] for HCA formation from a melt-prepared Na–(Ca)–Si–O–(P) glass exposed to SBF; the five HM stages are identified with the induction, proliferation, and maturation stages associated with sigmoidal growth (arrows; **left** panel). The first three HM steps involve (1) exchange of Na${}^{+}$/Ca${}^{2+}$ cations with protons from the solution, and (2) hydrolysis of Si–O–Si bonds, together leading to a high abundance of silanol (SiOH) surface groups, a portion of which (3) form Si–O–Si linkages by water removal. As depicted in the **right** panel, the HM stages (1)–(3) together produce a silica-gel layer, which comprises SiOH groups and water, but is nearly devoid of Na${}^{+}$/Ca${}^{2+}$ species. Next follows (4) a heterogenous nucleation of ACP, which then (5) crystallizes into HCA. The two last HM steps proceeds in parallel, with co-existing ACP/HCA components of the CaP layer (**bottom**, **right**), where HCA crystallizes from the interior of the ACP particles [14,15].

The five-step reaction mechanism accounting for the main events leading to HCA formation when a Na–Ca–Si–O–(P) glass is exposed to aqueous solutions was proposed by Hench [2] mainly on the basis of results from in vitro testing in (buffered) water [27,28,67]: while then *formally* only glasses that incorporate both Ca and phosphate species may produce HCA, the HM is also valid for in vitro testing in SBF media [56,57] as well as for real in vivo applications, for which the fluid surrounding the glass will provide the main Ca/P reservoirs of the biomimetic CaP layer. Below we recapitulate the five HM stages [2], assuming a modified (phospho)silicate glass exposed to a solution already comprising Ca and phosphate species, as illustrated schematically in Figure 4:

(1) First, a rapid exchange occurs of the Ca${}^{2+}$ and (particularly) Na${}^{+}$ cations with protons from the solution, where the glass-modifier leaching is accompanied by a rise in the pH of the solution, and creation of SiOH (“silanol”) groups at the glass surface.
(2) The silicate network at the surface fragments due to hydrolysis of Si–O–Si bonds according to Si–O–Si + H${}_{2}$O→2SiOH; this results in a further increased silanol concentration, but also in loss of Si species, which enter the solution as Si(OH)${}_{4}$.
(3) This step is essentially a reversal of stage (**2**), implying that Si–O–Si bonds form via condensation of neighboring SiOH moieties (see Figure 4).

The first three HM steps only involve reactions between silicate surface species and the surrounding solution. They altogether lead to a *hydrated silica gel* surface layer rich in SiOH groups but depleted in Ca${}^{2+}/$Na${}^{+}$ cations [2,28,29,30,41,72,81]; see Figure 4. As discussed further below, this SiO${}_{2}$-rich layer is believed to be pivotal for the in vivo/vitro bioactivity, by providing the structural sites triggering the heterogeneous CaP nucleation [29,30,41,72,81], with the last two HM steps proceeding as follows, where we first describe each stage by reproducing verbatim the Hench description [2]:

(4) “Migration of Ca${}^{2+}$ and PO${}_{4}^{3-}$ groups to the surface through the SiO${}_{2}$-rich layer forming a CaO–P${}_{2}$O${}_{5}$-rich film on top of the SiO${}_{2}$-rich layer, followed by growth of the amorphous CaO–P${}_{2}$O${}_{5}$-rich film by incorporation of soluble calcium and phosphates from solution” [2]; we remind that the “amorphous CaO–P${}_{2}$O${}_{5}$-rich film” is generally to be equated with “ACP”.
(5) According to Hench, the subsequent ACP→HCA transformation proceeds as “crystallization of the amorphous CaO–P${}_{2}$O${}_{5}$ film by incorporation of OH${}^{-}$, CO${}_{3}^{2-}$ or F${}^{-}$ anions from solution to form a mixed hydroxyl, carbonate, fluoroapatite layer” [2].

Notably, if *identifying the "amorphous CaP film/layer" with ACP*, its close correspondence to the well-established apatite precursor observed from spontaneous precipitation follows directly. Despite that this interpretation is already recognized by some researchers in the BG community and has experimental support (see Section 1), this view appears not to be wide-spread. Yet, in this (new) light, it is not surprising that HCA formed from silicate-based BGs in aqueous media manifests a sigmoidal growth (see Figure 3). Here, we may identify the first four HM steps as together constituting the induction period, while the final HM stage comprises both proliferation and maturation intervals. However, considering that stages 4 and 5 proceed largely simultaneously, one may also ascribe both of them as collectively accounting for the proliferation/maturation stages; these mappings between the HM steps and the sigmoid-growth stages are illustrated in Figure 4. We stress, however, that *some* ACP doubtlessly forms during the induction period, i.e., the fourth HM stage is limited but not absent.

The primary mechanistic distinctions between spontaneously precipitated ACP/HCA and their heterogeneously nucleated counterparts at a silicate-glass substrate involve the silicate-reaction events prior to ACP nucleation, after which an identical phenomenological HCA formation occurs. Moreover, the essentially identical nature of the ACP phase acting as precursor of both homogeneous/heterogeneous nucleation contexts suggest that they may *also* share the *same* detailed ACP→HCA conversion mechanisms. Yet, this proposal remains to be verified by future investigations.

The HM is generally supported by several studies of HCA formation from BGs and silica gels [30,31,32,33,37,40,41,42,43,47,54,82], yet with some deviations and/or refinements reported (besides those highlighted in the present article):

(*i*) A clear boundary between the two “silica gel” and “CaP” layers do not generally exist, where gradients of the Ca, P, and Si concentrations are usually observed outwards from the core of the glass [31,32,54,74]; CaP accumulation within the silica gel by Si–O–P bonding has also been proposed [29]. A minor CaP formation is occasionally reported at very early stages during BG immersion in buffered water/SBF [32,67], where Cerruti et al. [32] pointed out that several of the HM stages may proceed in parallel.

(*ii*) Except for stipulating that the ACP nucleation is triggered by the “silica gel” layer [2,28,29,30,41,72,81], the HM sheds no light on its precise role. Here, SiOH [81], Si–O${}^{-}$ [29,30,41], and trisiloxane [34,35] moieties have been suggested as potential nucleation sites. While the precise nucleation mechanisms differ, most proposals boil down to variations around the detailed reaction sequence introduced by Takadama et al. [41]: it initiates by proton release according to SiOH→SiO${}^{-}+$ H${}^{+}$, followed by association of the non-bridging oxygen ion of the SiO${}^{-}$ moiety with Ca${}^{2+}$ to yield an overall positively charged [SiO${}^{-}\cdots$Ca${}^{2+}$]${}^{+}$ complex that attracts phosphate anions to form CaP embryos that subsequently grow [41].

(*iii*) Stage 5 of the HM suggests that the incorporation of “foreign” ions (such as Na${}^{+}$, Mg${}^{2+}$, CO${}_{3}^{2-}$, and F${}^{-}$) only occurs in the apatite structure—i.e., subsequent to the ACP formation—while there are evidence from ${}^{23}$Na and ${}^{13}$C NMR that some Na${}^{+}$ and CO${}_{3}^{2-}$ ions are incorporated already into the ACP phase [37].

(*iv*) The large surfaces of sol-gel-derived BGs—and notably so MBGs with ordered arrangements of mesopores [46,55]—renders them inherently “gel-like” when exposed to aqueous solutions [37,43,83]. This feature accelerates the first three HM stages [37,43,47], thereby partially rationalizing the more rapid HCA formation observed from mesoporous BGs relative to their melt-prepared counterparts.

## 5. Concluding Remarks

On the basis of new experimental data from an SBF-exposed Na${}_{2}$O–CaO–SiO${}_{2}$–P${}_{2}$O${}_{5}$ bioactive glass in conjunction with existing literature results, we have contrasted the two prevailing but distinct understandings of HCA formation by nucleation from supersaturated solutions with that at BG substrates exposed to SBF solutions. Despite their adherence to the *formally distinct* classes of homogeneous and heterogenous nucleation, respectively, they exhibit many common aspects in their phenomenological apatite growth behavior, where we particularly highlight the following three features identified for HCA-growth from BG substrates (all of which are well-known in the context of spontaneously precipitated apatite):

(*i*) HCA forms via an ACP precursor with very similar (if not identical) chemical/structural nature as that preceding HCA formed from solutions supersaturated with respect to apatite. (*ii*) For increasing SBF-exposure intervals, the phenomenological apatite formation manifests the three-stage sigmoid-type growth of consecutive induction, proliferation, and maturation periods; however, solely the induction period is hitherto recognized and discussed in the literature on in vitro HCA formation from BGs because it reflects the “in vitro bioactivity” of the glass. (*iii*) The feature of “autocatalytic” apatite growth during the proliferation stage accounts for the majority of the apatite production, and for the SBF-exposed glass examined herein, implying ≈90% and ≈10% of the total HCA formation occurring during the respective proliferation and maturation stages.

Although the precise duration of each induction, proliferation, and maturation stage depends on the BG composition, the sigmoid functionality appears to hold generally for apatite grown in vitro from silicate-based glass surfaces, as witnessed by existing (qualitative) literature data on HCA formation in buffered water/SBF from BGs with widely differing compositions.

We also discussed our findings in view of the scheme proposed by Hench [2] for in vitro HCA formation from BGs. We observed largely *simultaneous* ACP/HCA formation processes, implying that a relatively modest (relative) amount of ACP forms during the induction interval, whereas the main portion emerges during the initial stages of the proliferation period, despite the rapid and concurrent ACP→HCA crystallization. This partially revises Hench’s mechanism by merging its last two events of as-proposed *consecutive* layer formations [2] of primarily amorphous CaP (step 4) and HCA (step 5). We conclude that the primary distinctions between the spontaneously precipitated CaP and its heterogeneous counterpart involving nucleation at a BG substrate solely concern the silicate-reaction events prior to ACP nucleation, after which an *identical phenomenological* HCA growth proceeds. Consequently, once the first embryos of ACP are nucleated—either homogeneously or heterogeneously—these scenarios might also share the same mechanistic details of the ACP→HCA transformation. These lines are worth exploring further, as they may unify the homogenous and heterogenous nucleation concepts and potentially reveal a universal apatite-growth model, while some aspects of the biomimetic apatite formation at BG surfaces may be learned from the comparatively more studied case of directly precipitated apatite.

## Supplementary Materials

The following are available online at http://www.mdpi.com/1996-1944/11/9/1690/s1, Figure S1: FTIR spectra shown across the full spectral range recorded.
