# Supplementary material for: Contrasting In Vitro Apatite Growth from Bioactive Glass Surfaces with that of Spontaneous Precipitation
Source: Materials (Basel). 2018 Sep 12;11(9):1690. doi: 10.3390/ma11091690 (PMC6164250; doi:10.3390/ma11091690)
Supplement: Supplementary file 1 [file materials-11-01690-s001.pdf]

Supporting Information for
Contrasting *In Vitro* Apatite Growth From Bioactive Glass Surfaces with that of Spontaneous Precipitation

Yang Yu, Zoltán Bacsik, and Mattias Edén*

Department of Materials and Environmental Chemistry, Stockholm University, SE-106 91 Stockholm, Sweden

*Corresponding author. E-mail: *mattias.eden@mmk.su.se*

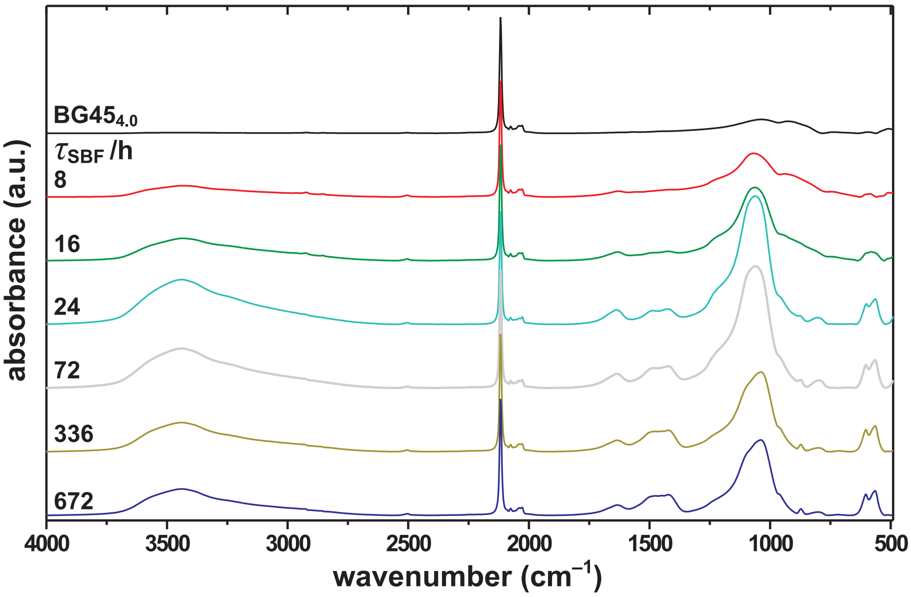

Fig. S1. FTIR spectra shown across the full spectral range recorded.
